# Response Surface Methodology Optimization of Ultrasonic-Assisted Extraction of *Acer Truncatum* Leaves for Maximal Phenolic Yield and Antioxidant Activity

**DOI:** 10.3390/molecules22020232

**Published:** 2017-02-04

**Authors:** Lingguang Yang, Peipei Yin, Hang Fan, Qiang Xue, Ke Li, Xiang Li, Liwei Sun, Yujun Liu

**Affiliations:** National Engineering Laboratory for Tree Breeding, College of Biological Sciences and Biotechnology, Beijing Forestry University, Beijing 100083, China; yanglingguangxdjqz@163.com (L.Y.); happy62889@126.com (P.Y.); fwqh1990@163.com (H.F.); xuetian20130607@163.com (Q.X.); like17931@163.com (K.L.); 18289743335@163.com (X.L.)

**Keywords:** *Acer truncatum* leaves, ultrasonic-assisted extraction, response surface methodology, phenolics, antioxidant activity, UPLC-QTOF-MS/MS

## Abstract

This study is the first to report the use of response surface methodology to improve phenolic yield and antioxidant activity of *Acer truncatum* leaves extracts (ATLs) obtained by ultrasonic-assisted extraction. The phenolic composition in ATLs extracted under the optimized conditions were characterized by UPLC-QTOF-MS/MS. Solvent and extraction time were selected based on preliminary experiments, and a four-factors-three-levels central composite design was conducted to optimize solvent concentration (*X*_1_), material-to-liquid ratio (*X*_2_), ultrasonic temperature (*X*_3_) and power (*X*_4_) for an optimal total phenol yield (*Y*_1_) and DPPH• antioxidant activity (*Y*_2_). The results showed that the optimal combination was ethanol:water (*v*:*v*) 66.21%, material-to-liquid ratio 1:15.31 g/mL, ultrasonic bath temperature 60 °C, power 267.30 W, and time 30 min with three extractions, giving a maximal total phenol yield of 7593.62 mg gallic acid equivalent/100 g d.w. and a maximal DPPH• antioxidant activity of 74,241.61 μmol Trolox equivalent/100 g d.w. Furthermore, 22 phenolics were first identified in ATL extract obtained under the optimized conditions, indicating that gallates, gallotannins, quercetin, myricetin and chlorogenic acid derivatives were the main phenolic components in ATL. What’s more, a gallotannins pathway existing in ATL from gallic acid to penta-*O*-galloylglucoside was proposed. All these results provide practical information aiming at full utilization of phenolics in ATL, together with fundamental knowledge for further research.

## 1. Introduction

*Acer truncatum* is a prominent maple (*Aceraceae*) species widely cultivated in China, Korea and Japan, and is also found in Europe and Northern America [1,2]. In northern China, maple leaves, mainly *A. truncatum* leaves (ATL), are often used as health drinks and folk medicines for treating coronary artery cirrhosis, cerebrovascular diseases and angina pectoris [3]. Previous investigations of ATL indicated that it possessed various biological functions, such as antioxidant [4], antibacterial [5,6], and antitumor properties [7,8] due to its high contents of tannins, flavonoids, and chlorogenic acid. However, little effort has been made to optimize of methods of extraction of phenolics from ATL. Therefore, in order to ensure the full utilization of ATL, in the current study an ultrasound-assisted extraction (UAE) method was established through response surface methodology (RSM).

UAE is well known to display positive effects on the rate of various extraction processes used in the food, pharmaceutical and cosmetic industries. Compared to other conventional methods, UAE offers a net advantage in terms of productivity and selectivity with shorter processing times, enhanced quality, reduced chemical and physical hazards, and it is environmentally friendly and causes less damage to the structural and molecular properties of compounds in plant materials [9,10]. For these reasons, UAE from plant materials has been widely used lately to facilitate extractions of phenols from *Chrysanthemum morifolium* flower heads [10], *Olea europaea* fruits [11], *Morus nigra* must [12], and *Curcuma longa* rhizomes [13], lipids from rice [14], polysaccharides from pumpkin [15] and mycelial fermentation of *Phellinus igniarius* [16], proteins from *Jatropha curcas* seeds [17], lignans from *Schisandra chinensis* fruits [18] and other value-added compounds from various natural resources. However, to the best of our knowledge, no report is available regarding improvements of phenolic yield by itself, let alone the phenolic yield together with antioxidant activity from ATL by using ultrasonic stimulation. Thus the present study could provide useful information for the industrial manufacture of ATL as a phenolic and antioxidant resource.

Being a valuable tool to investigate the interaction among factors and quantitatively depict the effects of given parameters on their measured responses [19], RSM is a collection of statistical and mathematical methods for developing, improving, and optimizing a process [20]. In comparison with single variable optimization methods, RSM is a time- and cost-effective means of simultaneously evaluating interactions as well as the key experimental parameters [21], thus have been wildly applied to optimize many bioprocesses [17,22,23,24]. Central composite design (CCD) used in RSM, is superior compared with the classical approach in terms of the comprehensiveness of information gained and the accuracy of the experiments conducted [22,25], thus is applied in the present study to optimize the UAE process.

The LC-MS/MS technique is gaining increasing interest in the characterization of phenolic components due to its high selectivity and sensitivity [26,27,28]. For example, Melguizo-Melguizo et al. [29] analyzed 22 compounds from *Artemisia vulgaris* leaves, 15 of them were phenolics; and Kolniak-Ostek [30] tentatively identified 65 phenolic components in ten pear cultivars. Therefore, the variety of phenolics in ATL extract, determined with UPLC-QTOF-MS/MS analysis under the optimized extraction condition, was analyzed to investigate the phenolic composition as well as the quality of the extraction.

Overall, the present study was designed to optimize the UAE process with RSM and CCD for the maximum phenolic yield and antioxidant activity from ATL. The phenolic composition of the ATL extract obtained under the optimized UAE conditions was then analyzed with the UPLC-QTOF-MS/MS technique. These results should contribute to the baseline data for industrial manufacture as well as further exploitation of ATL as a phenolic and antioxidant compound source.

## 2. Results and Discussion

### 2.1. Effects of Solvents and Independent Variables on UAE

#### 2.1.1. Solvent Types

**Figure 1:**
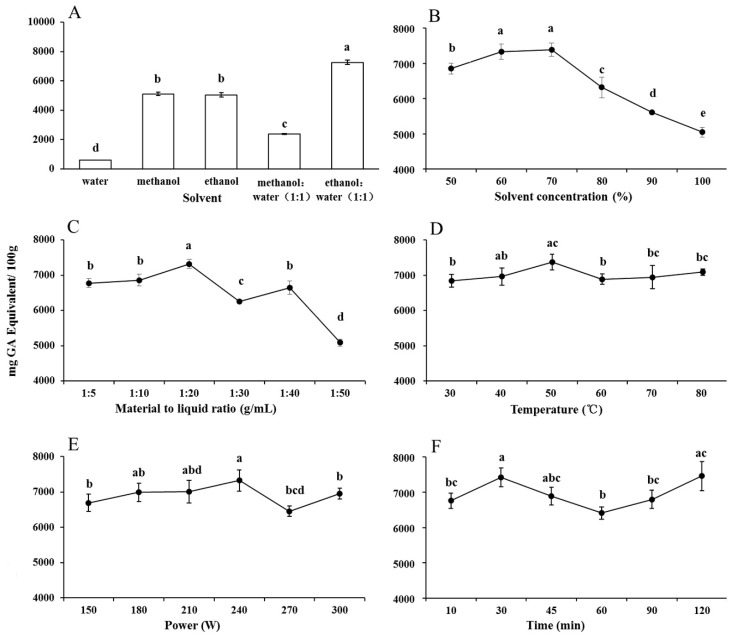
The effect of solvent type (**A**) and solvent concentration (**B**) extracted at 240 W, 50 °C and 30 min, with material to liquid ratio at 1:20 g/mL, material to liquid ratio (**C**, extracted at 240 W, 50 °C, 30 min, and 70% aqueous ethanol), extraction temperature (**D**, 240 W, 30 min, 70% aqueous ethanol, and 1:20 g/mL), sonication power (**E**, 50 °C, 30 min, 70% aqueous ethanol, and 1:20 g/mL), and extraction time (**F**, 240 W, 50 °C, 70% aqueous ethanol, and 1:20 g/mL) on the yields of total phenols in ATL by single factor test. Extraction was repeated thrice. Values marked by the same letter are not significantly different (*p* < 0.05).

The phenolic yield obtained with different extraction solvents (water, methanol, ethanol, 1:1 (*v*:*v*) methanol:water, or 1:1 (*v*:*v*) ethanol:water) was determined first to select the best solvent (combination). The results showed that different solvents significantly affected the phenolic yield (Figure 1A). The phenolic yield with methanol (5112.17 GAE/100 g d.w.) was slightly higher than that with ethanol (5049.23), and both of them were significantly higher than that with water (602.63). However, when ethanol was combined with water at the ratio of 1:1 (*v*:*v*), the phenol extraction was significantly increased to 7262.61. This result was consistent with several previous studies reporting that aqueous organic solvents exhibit a higher extraction efficiency than absolute organic solvents [31,32,33]. Cujic et al. [34] explained this phenomenon by the fact water is responsible for swelling of the plant material while the ethanol is responsible for disrupting the bonding between the solutes and the plant matrix, and this synergistic effect leads to a higher phenolic yield. However, the phenolic yield obtained with 1:1 (*v*:*v*) methanol:water was worse (2369.13) compared with that extracted merely with methanol. This might result from its inappropriate polarity for extractions targeting phenols in ATL. Therefore, ethanol but not methanol, and aqueous ethanol, instead of pure ethanol, was selected as the best solvent for phenolic extraction.

#### 2.1.2. Solvent Concentration

Aqueous ethanol of different concentrations was used to conduct the single factor analysis under the following uniform conditions for material-to-liquid ratio, extraction temperature, ultrasonic power, and extraction time: 1:20 g/mL, 50 °C, 240 W, and 30 min, respectively. As shown in Figure 1B, phenolic yield increased from 6850.04 mg GAE/100 g d.w. to 7329.02 for ethanol concentrations between 50% and 60%, and to the maximum (7388.07) at 70%. The efficiency then decreased with further increases in the ethanol concentration, and reached the minimum (5026.35) at 100%. Therefore, it can be concluded that aqueous ethanol exhibited the closest polarity to the phenols in ATL at the range between 60% and 80% as the efficiency reached the maximum value with ethanol concentration at 70%. Thus, the variable range of ethanol concentration used in the RSM experiments was 60%–80%.

#### 2.1.3. Material-to-Liquid Ratio

Phenolic yields with different material-to-liquid ratios were evaluated in the single factor analysis under the following uniform conditions for aqueous ethanol concentration, extraction temperature, ultrasonic power, and extraction time: 70%, 50 °C, 240 W, and 30 min, respectively. As shown in Figure 1C, phenolic yield increased slightly from 6772.95 mg GAE/100 g d.w. to 6858.25 at ratios between 1:5 and 1:10, and much rapidly to the maximum (7320.00) at 1:20. The efficiency declined at ratios between 1:20 and 1:30, then increased to a second peak at 1:40 and reached the minimum (5086.68) at 1:50. Based on the mass transfer principles occurred in UAE, the driving force for mass transfer is considered to be the concentration gradient between the solid and solvent [35], as well as the partition coefficient of phenolics between the natural matrix and the solvent [36]. These results revealed that the optimal ratio leading to the strongest driving force was 1:20 g/mL for phenolic extraction in ATL. Thus, the variable range of material-to-liquid ratio used in the RSM experiments was determined as 1:15–1:25 g/mL.

#### 2.1.4. Extraction Temperature

To study the effect of extraction temperature on phenolic yield, UAE was implemented at different temperatures under the following uniform conditions for ethanol concentration, material-to-liquid ratio, ultrasonic power, and extraction time: 70%, 1:20 g/mL, 240 W, and 30 min, respectively. As shown in Figure 1D, phenolic yield increased from the minimum (6840.00 mg GAE/100 g d.w.) at 30 °C to the maximum (7376.17) at 50 °C, decreased to 6890.21 at 60 °C, then slightly increased up to 80 °C with no significant differences thereafter between 60 and 80 °C. These results suggest that a relative high temperature increased phenolic extraction efficiency as it increased the number of cavitation nucleus formed [37], leading an enhanced mass transfer and therefore a better access of solvent to cell components [38]. However, this increase declined, namely, brought a negative effect on the efficiency when the temperature exceeded a certain value. This was mainly due to the decreased cavitation intensity under increased temperatures [39,40]. In addition, the accelerated evaporation would also be induced at high temperatures, which led to the decrease of driving force [41]. A temperature effect on yield with the same pattern in a UAE process was previously reported, such as in the cases of phenols from olive fruit [11], oil from pomegranate seed [41], and carotene from citrus peels [42]. Thus, an extraction temperature ranging from 40 to 60 °C was chosen in the RSM process of ATL.

#### 2.1.5. Ultrasonic Power

The effect of ultrasonic power on phenolic yield was determined under the following uniform conditions for ethanol concentration, material-to-liquid ratio, extraction temperature, and extraction time: 70%, 1:20 g/mL, 50 °C, and 30 min, respectively. As shown in Figure 1E, phenolic yield increased from 6693.94 mg GAE/100 g d.w. at 150 W to the maximum (7327.12) at 240 W. The yield decreased to the minimum (6456.36) at 270 W, then increased to 6959.13 at 300 W. There was no significant difference between 150, 180, and 210 W or between 270 and 300 W. According to a report by Hemwimol et al. [43], when ultrasonic waves with larger amplitude travel through an extraction solvent, more bubbles would be created, and more collapse and violent shockwaves and high-speed jets might be generated to disrupt the cell walls. Therefore, the penetration of extraction solvent into cells became stronger, resulting in more release of target components from cells into the mass medium. Consequently, the mass transfer rate was thus enhanced and this led to an appropriate increase in yield. However, the extraction efficiency tended to decrease when the ultrasonic power was higher than 240 W, and this might be caused by degradation of bioactive compounds and overproduced bubbles which could hamper the propagation of ultrasound waves at too high an ultrasonic power [44]. Thus, an ultrasonic power range from 210 to 270 W was chosen in the RSM process of ATL.

#### 2.1.6. Extraction Time

The effect of extraction time on phenolic yield was determined under the following uniform conditions for ethanol concentration, material-to-liquid ratio, extraction temperature, and ultrasonic power: 70%, 1:20 g/mL, 50 °C, and 240 W, respectively. As shown in Figure 1F, a biphasic increase in phenolic yield was observed with the increase of the extraction time. Phenolic yield increased from 6760.65 mg GAE/100 g d.w. to 7421.41 when the sonication time was prolonged from 10 to 30 min, then declined to the lowest value (6411.25) at 60 min, and the yield increased again to the maximum (7458.61) at 120 min. There was no significant difference between phenolic yields at 10 and 30 min. The sonication time was recognized as a significant factor increasing the extraction efficiency of UAE processes, however, the extraction efficiency decreased in some cases due to a prolonged sonication time as it will lower the permeability of solvent into the cell walls due to oversuspended impurities [14]. Moreover, prolonged extraction time may increase the chances of decomposition of phenolics [45] and also potentially increase the loss of solvent by vaporization [46], which can directly affect the loss of mass transfer during extraction. Considering energy-savings, 30 min was chosen to conduct the experimental design tests described below.

### 2.2. Statistical Analysis and Model Fitting

To optimize the operating parameters, 30 random sequential experiments were performed under the designed UAE conditions based on the ranges of every single factor (independent variable) determined above to study the reciprocal influence of independent variables (i.e., solvent concentration, material-to-liquid ratio, extraction temperature and ultrasonic power) on the two dependent (response) variables (i.e., phenolic yield and its corresponding antioxidant activity). The experimental values, together with predicted values obtained by their response surface central composite design, are presented in Table 1.

The final quadratic equation obtained in terms of actual factors upon applying multiple regression analysis to the experimental data is given below:

When the phenolic extraction efficiencies (*Y*_1_) were considered as the response:

$$Y_{1}=\;-9574.12+322.61X_{1}+126.39X_{2}+43.31X_{3}-4.91X_{4}+1.51X_{1}X_{2}-0.03X_{1}X_{3}+0.31X_{1}X_{4}-0.16X_{2}X_{3}-1.13X_{2}X_{4}+0.24X_{3}X_{4}-2.76X_{1}^{2}-3.51X_{2}^{2}-0.10X_{3}^{2}-0.44X_{4}^{2}$$

When the antioxidant capacities (*Y*_2_) were considered as the response:

$$Y_{2}=\;-78025.43+1807.32X_{1}+1586.92X_{2}+15.99X_{3}+713.92X_{4}-1.75X_{1}X_{2}+0.78X_{1}X_{3}-1.12X_{1}X_{4}-1.31X_{2}X_{3}-11.16X_{2}X_{4}+1.72X_{3}X_{4}-14.22X_{1}^{2}-15.76X_{2}^{2}-0.27X_{3}^{2}-7.95X_{4}^{2}$$

where, *X*_1_, *X*_2_, *X*_3_, *X*_4_, *Y*_1_, *Y*_2_ are the aqueous ethanol concentration, material-to-liquid ratio, extraction temperature, ultrasonic power, phenolic yield response and antioxidant activity response, respectively.

The linear effect of solvent concentration (*X*_1_) was found to be significant for both response variables while *X*_4_ was only significant for phenol yield (*Y*_1_), and *X*_2_ and *X*_3_ were only significant for antioxidant activity (*Y*_2_). It can be concluded that solvent concentration was the vital parameter in both responses. However, the quadratic effect (*X*_1_^2^, *X*_2_^2^, *X*_3_^2^) was found to produce extremely significant (*p* < 0.01) positive effect on both *Y*_1_ and *Y*_2_, but *X*_4_^2^ was only significant for *Y*_2_.

ANOVA results for multiple regression analysis and response surface quadratic model of *Y*_1_ and *Y*_2_ were evaluated using the corresponding *F* and *p* values (Table 2). *F* values of *Y*_1_ and *Y*_2_ were calculated to be 25.95 and 50.34, both leading to a *p* value < 0.0001, suggesting that both the models were statistically extremely significant. The models’ coefficient of determination (*R*^2^) were 0.9604 and 0.9792, indicating that more than 96.04% and 97.92% of the response variability is explained, and supporting a good accuracy and ability of the established model within the range limits used [47]. Correlation coefficients of 0.9800 and 0.9898 also indicate a good positive correlation between the actual data and the predicted values obtained using CCD. The *F*-values of Lack of Fit of *Y*_1_ and *Y*_2_ were 0.6032 and 0.2015, respectively, implying that the Lack of Fit is not significant relative to the pure error, thus the model can be used to predict the phenol yield and corresponding antioxidant activity of ATL. In addition, Adj-*R*^2^, Pre-*R*^2^ and the coefficient of variation (C.V.) were calculated to check the model’s adequacy. Pre-*R*^2^ of *Y*_1_ and *Y*_2_ were 0.8341 and 0.8968, which were in reasonable agreement with their Adj-*R*^2^ of 0.9233 and 0.9597, respectively (Adj-*R*^2^ − Pre-*R*^2^ < 0.2), indicating a high degree of correlation between the measured and predicted data from the regression model [48]. The C.V. expressed the standard deviation as a percentage of the mean, and was found to be 1.5169% (<5.00%) for the phenolic yield, and 2.3491% (<5.00%) for antioxidant activity, implying that the models were reproducible [24]. Adequate precision measures the signal to noise ratio, which is desirable when the value is larger than 4. 

The ratios of 22.3775 and 28.1801 both referred an adequate signal and illustrated that the models (*Y*_1_ and *Y*_2_) were applicative for the present UAE process [49]. These correlation analyses between predicted values and actual data can be used to evaluate the suitability of the response surface model [50]. Thus, the model can be used to predict the phenolic yield and corresponding antioxidant activity under various extraction conditions during UAE process. As shown in Table 1, the levels of phenolic yield ranged from 5783.04 to 7591.30 mg GAE/100 g d.w., and the levels of antioxidant activity ranged from 42,030.84 to 73,849.78 μmol TE/100 g d.w. The highest levels of phenolic yield (7256.52 to 7587.83 mg GAE/100 g d.w.) and antioxidant activity (70,844.41 to 73,849.78 μmol TE /100 g d.w.) were obtained under the center point combinations of 70% ethanol, material-to-liquid ratio of 1:20 g/mL, 240 W, 50 °C, and 30 min. Moreover, the conditions involving a solvent concentration of 66.21%, material-to-liquid ratio of 1:15.31 g/mL, ultrasonic temperature 60 °C, power 267.30 W, and extraction time of 30 min were predicted to provide the highest phenolic yield, together with the highest antioxidant activity according to the fitted models. Ratios of DPPH activity/total phenols were calculated and we found that these ratios ranged between 7.29 and 9.91, which means the phenolic components extracted in all 30 runs were similar. Moreover, the ratios of six repetitions with the condition at the central point (9.60–9.91) were higher than those of the others, further indicating that the antioxidant activity of phenolics extracted under the condition represented by the central point was superior and the results of single factor experiments were reliable. Total phenols of the leaves collected in April optimized with UAE in the present study was lower than those (93.08 mg/g, i.e., 9308 mg/100 g) of fallen leaves reported by Cai et al. [51] prepared with a microwave method. However, according to another study conducted by ourselves [52], *Acer truncatum* fallen leaves naturally possessed a much higher total phenols and radical scavenging activity than those collected in other seasons such as in April. Considering the difference in sampling seasons mentioned above, the data discrepancy between our two studies might be considered to be within a reasonable range.

To further verify the models obtained from RSM, ATL was extracted under the predicted optimal UAE conditions, and its phenolic yield and antioxidant ability were evaluated and compared to the predicted maximum yield. For operational convenience, the optimal parameters were modified slightly in the verification experiment as follows: solvent concentration 66.20%, material-to-liquid ratio 1:15.30 g/mL, ultrasonic power 270.00 W, temperature 60 °C, and extraction time 30 min. The predicted phenolic yield and antioxidant activity under the optimal conditions were 7589.19 mg GAE/100 g and 74,010 μmol TE/ 100 g, and the experimental values under the optimal conditions were 7579.56 ± 354.44 mg GAE/100 g and 73,585.78 ± 790.74 μmol TE/100 g, respectively. No significant differences were observed between predicted and experimental values (*p* > 0.05), indicating that the experimental results confirmed the adequate fitness of the predicted model.

### 2.3. Effect of Interactions Among Variables on Phenolic Yield and Antioxidant Activity in ATL

**Figure 2:**
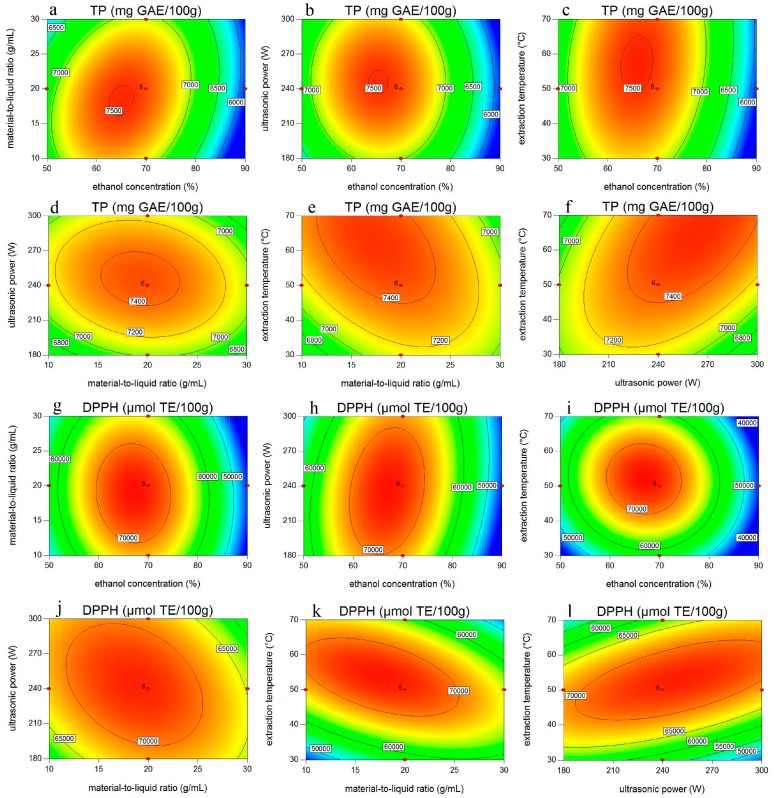
Contour plots showing the effects of four variables (solvent concentration, material to liquid ratio, ultrasonic power, and extraction temperature) and their interactions on extraction efficiency of total phenols (TP, **a**–**f**) and their DPPH• scavenging capacity (**g**–**l**). The other two variables in each of the Figures were kept constant at their respective central experimental values.

To visualize the interactions of two operational parameters on extraction efficiency, the responses were generated as planar contour plots (Figure 2). Two variables unshown in the Figures were kept constant at their respective central experimental values and the other two variables presented on the two horizontal axis varied within their experimental ranges in order to understand their main and interactive effects on the dependent variables. Figure 2a–f show the results of interactive influence of solvent concentration, material-to-liquid ratio, extraction temperature, and ultrasonic power on phenolic yield, while Figure 2g–h exhibit the impact of these variables on antioxidant activity.

Figure 2a–c, g–i show the phenols and antioxidant activities as responses of aqueous ethanol concentration (*X*_1_) and the other factors (*X*_2_, *X*_3_, *X*_4_). The extraction efficiencies first increased then decreased with increase of aqueous ethanol concentration from 50% to 90%, material-to-liquid ratio from 1:10 to 1:30 g/mL, ultrasonic power from 180 to 300 W, and extraction temperature from 30 to 70 °C.

The interaction of solvent concentration and material-to-liquid ratio (*X*_1_*X*_2_) showed an extremely significant positive effect (*p* < 0.001) on both responses (see Table 2). At lower ethanol concentration with increasing material-to-liquid ratio, total phenols and antioxidant activity kept generally mild. However, the total phenols with antioxidant activity first increased, then decreased at lower material-to-liquid ratio with increasing ethanol concentration (Figure 2a,g).

The possible interaction mechanism between solvent concentration and material-to-liquid ratio might be interpreted as that the positive interaction caused by appropriate solvent concentration and material-to-liquid ratio affected the polarity and viscosity of aqueous ethanol and solubility of target phenolic compounds in the extraction solvent, thus influenced the yield of phenols.

As shown in Figure 2f,l, the interaction between sonication power and temperature (*X*_3_*X*_4_) also showed a significant positive effect (*p* < 0.05) on total phenols and antioxidant activity (Figure 2f). The possible interaction mechanism between temperature and power may be the change of cavitation threshold affected by changing temperature, which is responsible for acoustic cavitation and also results in the formation of a cavitational nucleus. The influence of relatively greater forces ruptures and erupts the formed cavitational nucleus and disrupts the cell tissues during extraction, which in turn enhances the mass transfer rate [38].

With respect to antioxidant activity (*Y*_2_), the interaction effect of solvent concentration and sonication power (*X*_1_*X*_4_) was also found to be significant (*p* < 0.05). According to the study of Hemwimol et al. [43], only a small fraction of the electric energy from the ultrasound actually entered the extraction solvent in the ultrasonic bath system, and most of it was absorbed by the water in the bath. Under this circumstances, the rise of the solvent concentration might increase the utilization efficiency of the limited electric energy, thus playing a vital role in the improvement of the extraction yield.

### 2.4. Characterization of Phenolic Compositions in ATL

**Figure 3:**
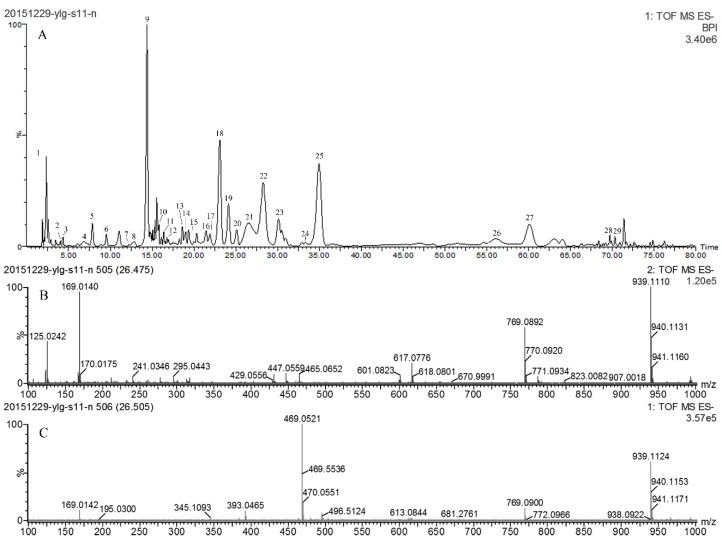
UPLC-QTOF-MS/MS data of the ATL extract obtained under the optimized conditions. Besides the UPLC-MS profile (**A**), a peak with the retention time at 26.51 min was identified as pentagalloyl glucose based on its MS (**B**) and MS/MS (**C**) fragments.

Table 3 shows a list of 29 phenolic compounds identified in ATL prepared under the optimal extraction conditions obtained above through UPLC–QTOF-MS/MS experiments, along with their retention times (RT), experimental *m/z*, calculated *m/z*, error values (ppm), molecular formula and MS/MS fragments. As signals in negative mode was stronger than those in the positive mode, data collected in negative mode was thus chosen to conduct the identification. Mass spectra in negative ion mode (Figure 3A), together with MS (Figure 3B) and MS/MS (Figure 3C) fragments of the peak with retention time at 26.51 min were presented as follows.

#### 2.4.1. Gallates and Gallotannins Derivatives

Compound **2** (*R*_t_ = 4.03 min) was identified as theogallin (a galloylquinic acid) with a [M − H]^−^ ion at *m/z* 343.0654. It showed a major fragment at *m/z* 191 (quinic acid) due to the loss of a galloyl moiety (152 amu) and another fragment at *m/z* 169 (gallic acid) due to the loss of a part of the quinic acid moiety (174 amu). Compound **3** (4.29 min) gave a [M − H]^−^ ion at 169.0143 and a major fragment ion at *m/z* 125 caused by the loss of a -CO_2_ group, and was identified as gallic acid. Compound **4**, with a [M − H]^−^ ion at *m/z* 183.0323 and a major fragment ion at *m*/*z* 124 due to the loss of -CO_2_CH_3_ (59 amu) was assigned as methyl gallate. The parent ions and MS/MS profiles of these three gallates accorded with the data reported in the literature [53].

Moreover, the gallic acid ion at *m/z* 169 occurred not only in compounds **2** and **3**, but also in compounds **16**, **21**, **22**, and **26**, indicating that all these compounds share the gallic acid moiety. Compound **16** (21.47 min) with a deprotonated ion at *m/z* 787.1001 was identified as tetra-*O*-galloyl-glucoside. It fragmented at *m/z* 615 with a loss of one gallic acid moiety (170), and fragmented at *m/z* 465 with another loss of a galloyl unit (152). Meanwhile, compound **21** gave a [M − H]^−^ ion at *m/z* 939 with the molecular formula C_41_H_32_O_26_. It fragmented at *m/z* 769 with a loss of a gallic acid moiety (170), and fragmented at *m/z* 617 with another loss of galloyl (152) (Figure 3B,C). Thus compound **21** (26.51 min) was identified as a penta-*O*-galloylglucoside isomer. With the same parent ion and fragments, compound **22** (28.23 min) was identified as another penta-*O*-galloylglucoside isomer. One of them should be the 1,2,3,4,6-pentakis-*O*-galloyl-β-d-glucose, previously identified in ATL [54]. Compound **26** (56.11 min) with a deprotonated ion at *m/z* 1091.12 was identified as hexa-*O*-galloyl-glucoside. It fragmented at *m/z* 939 and 769 following the mode described above. The parent ions and fragmentation mode of these gallotannins were reported in the literature [55].

Accordingly, we hypothesized that a gallotannins pathway exists in ATL, leading to the biosynthesis of tetra-*O*-galloylglucoside, penta-*O*-galloylglucoside and hexa-*O*-galloylglucoside from gallic acid. Grundhöfer et al. [56] elucidated a pathway forming complex gallotannins in *Rhus typhina* through enzyme studies. They reported the detailed conversion process from gallic acid to β-glucogallin, digalloyglucose, trigalloyglucose, tetragalloyglucose, pentagalloylglucose, and hexagalloyglucose. As most of galloylglucoses mentioned above were also found in ATL, we conjecturethat the gallotannins pathway existing in ATL might be quite similar with the one in *Rhus typhina*. In addition, 1,2,3,4,6-pentakis-*O*-galloyl-β-d-glucose was the common precursor of two subclasses of hydrolyzable tannins, the gallotannins and the related ellagitannins [53]. Thus the pathway from gallic acid to penta-*O*-galloylglucoside might be the tip of the iceberg. Unfortunately, gallotannins with a higher degree of polymerization than that of hexagalloyglucose were not found in the present study.

#### 2.4.2. Flavonoids

Compound **29** (71.72 min) with a parent ion at *m/z* 301.0354 was identified as quercetin by comparison with the MS/MS data of a previous study [57]. Moreover, the quercetin moiety as a daughter ion at *m/z* 301 also existed in compounds **10**, **15**, **19**, **20**, **23**–**25**, and **28**, from which the abundance of quercetin derivatives in ATL could be inferred. Compound **10** (16.08 min) was assigned as quercetin 3-rhamninoside as its molecular ion was at *m/z* 755.2031 [58]. It fragmented at *m/z* 301 (quercetin) as its aglycone ion, and fragmented at *m/z* 609 due to the loss of a rhamnosyl (146 amu), and fragmented at *m/z* 463 due to another loss of a rhamnosyl. Compound **15** (19.38 min) was identified as quercetin-3,7-*O*-alpha-l-dirhamnopyranoside with a [M − H]^−^ ion at 593.1513 and a daughter ion at 447 caused by the loss of a rhamnosyl (146 amu) [59]. According to Lin and Harnly [60], the glycosylated quercetins with a monosaccharide at the same position elute from C_18_ columns in the following order: galactoside, glucoside, xyloside, arabinopyranoside, arabinofuranoside, rhamnoside and glucuronide, and this order was further confirmed by Keinänen and Julkunen-Tiitto [61]. Thus compounds **19** (24.15 min), **20** (25.09 min), **23** (30.12 min), **24** (33.32 min), **25** (34.94 min), and **28** (69.26 min), with parent ions at *m/z* 463.0877, 463.0884, 433.0768, 433.0767, 447.0927, and 609.1234, respectively, were identified as quercetin-3-*O*-galactoside (hyperoside), quercetin-3-*O*-glucoside (isoquercetin), quercetin-3-*O*-arabinopyranoside, quercetin-3-*O*-arabinofuranoside (avicularin), quercetin-3-*O*-rhamnoside (quercitrin), and quercetin-3-*O*-rutinoside (rutin), respectively. Furthermore, by comparing their parent ions and fragments with those of literatures, the above deductions on compounds **19** [57], **20** [57], **25** [53], and **28** [59] were verified.

Compounds **13** and **14** shared a parent ion at *m/z* 479, and their common daughter ion at *m/z* 316 was assigned as a myricetin moiety. According to Heras et al. [28], compounds **13** (18.65 min) and **14** (19.01 min) were identified as myricetin-*O*-hexoside I and myricetin-*O*-hexoside II, respectively. Besides compounds **13** and **14**, compounds **17** and **18** also shared the myricetin moiety ion at *m/z* 316, thus they were considered myricetin derivatives. Compound **17** (21.95 min) with molecular ion at *m/z* 449.0727 was identified as myricetin-arabinoside/xylopyranoside as its MS/MS profile corresponded with that of the literature [27]. Compound **18** (23.10 min) was assigned as myricetrin-3-*O*-rhamnoside (myricitrin) with a [M − H]^−^ ion at *m/z* 463.0878 and an aglycone ion at *m/z* 316 [62].

Compound **11** (16.39 min) was identified as catechin, as its molecular ion was observed at *m/z* 289.0717. It fragmented at *m/z* 245 and 211, corresponding with its MS/MS data in the literature [26]. Compound **27** (60.30 min) possessed a deprotonated ion at *m/z* 431.0974, and was identified as kaempferol-3-*O*-rhamnoside [59]. Its fragment ion at 285 was assigned as the kaempherol moiety.

#### 2.4.3. Chlorogenic Acid Derivatives

Compound **1** (2.35 min) with deprotonated ion at *m/z* 191.0564 was identified as quinic acid by comparing its MS/MS data with the literature [29]. Quinic acid is a cyclic polyol, instead of a phenol itself, however, it is an important part of caffeoylquinic acid derivatives. Compounds **4** (6.91 min) and **5** (7.85 min) were found to share a deprotonated ion at *m*/*z* 353, indicating the presence of monocaffeoylquinic acid isomers. According to Ncube et al. [63] and Melguizo-Melguizo et al. [29], the common base peak in their MS/MS spectra at *m/z* 191 illustrated the presence of a quinic acid moiety, and the fragment ion at *m/z* 179 occurring in compound **4** indicated that caffeoyl group is linked to the 3-OH position of quinic acid. Therefore, compound **4** was identified as 3-*O*-caffeoylquinic acid, while compound **5** was identified as 5-*O*-caffeoylquinic acid due to the different fragmentation pattern.

Compounds **7** (12.64 min) and **8** (12.93 min) were identified as *p*-coumaroylquinic acid isomers as they shared a deprotonated ion at *m/z* 337. According to Ncube et al. [63], *trans*- and *cis*-5-*p*-coumaroylquinic acids possess a base peak at *m/z* 191, while *trans*- and *cis*-4-*p*-coumaroylquinic acid possess one at *m/z* 173. As they shared a base peak at *m/z* 191 in the present study, and considering their reported eluting order, compounds **7** and **8** were identified as *trans*- and *cis*-5-*p*-coumaroylquinic acids, respectively.

#### 2.4.4. Other Phenolic Compounds

Compound **6** (11.05 min) with a molecular ion at *m/z* 285.0612 was identified as uralenneoside as it fragmented at *m/z* 153 due to the loss of a hydroxybenzoic acid unit and at *m/z* 109 because of another loss of a CO_2_ group from the carboxylic acid moiety, and its MS/MS data were accordance with the fragmentation pattern reported by Yu et al. [64]. Compound **12** (16.91 min), with a deprotonated ion at *m/z* 863.1819, was identified as cinnamtannin B1 as it consisted of three epicatechin units, which was reflected by the daughter ion at *m/z* 289 [65].

To sum up, the 29 phenolics identified consisted of seven gallate and gallotannin derivatives, 15 flavonoids (most flavonol-3-*O*-glycosides), five chlorogenic acid derivatives, and two other phenolic compounds. Previously, seven phenolic compounds were identified in ATL as gallic acid, quercetin, quercetin-3-arabinopyranoside [66], cholorogenic acid [3], methyl gallate [4], quercetin-3-*O*-l-rhamnoside [3], and 1,2,3,4,6-penta-*O*-galloyl-β-d-glucose [67]. To the best of our knowledge, the other 22 phenolics were firstly discovered in the present study. These findings provided the fundamental information to characterization of the phenolic compositions in ATL, and signified the superiority of the UAE condition optimized to a certain extent.

## 3. Materials and Methods

### 3.1. Chemicals and Plant Materials

Folin-Ciocalteu reagents, 2,2-diphenyl-1-picrylhydrazyl (DPPH), and 6-hydroxy-2,5,7,8-tetramethylchroman-2-carboxylic acid (Trolox) were purchased from Sigma Chemical (St. Louis, MO, USA). HPLC grade acetonitrile and formic acid were purchased from Fisher Scientific (Pittsburgh, PA, USA). Ultra-pure water was prepared using a Milli-Q50 SP Reagent Water System (Millipore Corporation, Billerica, MA, USA). Other reagents (analytical grade) were purchased from Sinopharm Chemical Reagent Co. Ltd. (Beijing, China).

Twenty *Acer truncatum* Bunge trees, whose tree-age (around 15 years old) and growth environment were approximatively identical, were randomly selected in Bajiajiaoye Park, Haidian District (N 40°00′57.21′′; E 116°19′43.36′′ with altitude 39–41 m) and authenticated by associate professor Zhonghua Liu, Beijing Forestry University, Beijing, China. *A. truncatum* leaves (ATL) were uniformly collected from the selected trees in 20 April 2015 and taken back to the laboratory immediately. The leaves were cleaned with distilled water and air-dried, the dried leaves were ground and passed through a 250 × 250-μm sieve, and the powder was stored at −20 °C in a refrigerator for extraction.

### 3.2. Optimization of UAE

#### 3.2.1. Preliminary Experiments

To test the impact of solvent type on phenolic yield, 1.000 g leaf powder was mixed thoroughly with different solvents, including water, methanol, ethanol, methanol: water (1:1, *v*:*v*) or ethanol:water (1:1, *v*:*v*) at a material-to-liquid ratio (g/mL) of 1:20 in a plastic centrifuge tube. The tube was then immersed into a tunable ultrasonic cleaning bath (KQ-300DE type, Kunshan Ultrasonic Instrument Co., Ltd., Kunshan, China) with the liquid level in the tube kept lower than that of the cleaner tank, and extracted under ultrasonic conditions at 50 °C, 240 W and 30 min. The sample mixture was then centrifuged for 10 min at 6000 rpm and the supernatant was collected. The resulting residue were repeated for extraction twice more with the same volume of solvent under the specific material-to-liquid ratio, and all the supernatants were combined, filtered, and diluted to a final volume of 60 mL. The resulting solutions were analyzed for total phenols. The optimal solvent with the highest total phenol content was selected for the following experiments.

The effect of each independent variable on phenolic yield was determined by single factor experimental designs. ATL was extracted with the optimal solvent selected and different concentrations (50%, 60%, 70%, 80%, 90% and 100%), material-to-liquid ratios (1:5, 1:10, 1: 20, 1:30, 1:40, 1:50 g/mL), ultrasonic powers (150, 180, 210, 240, 280 and 300 W), sonication temperatures (30, 40, 50, 60, 70 and 80 °C) and sonication time (10, 30, 45, 60, 90 and 120 min). As described above, the starting mass was always 1.000 g leaf powder, when different material-to-liquid ratios is applied, the final volume changes correspondingly (e.g., the final volume would be 90 mL if the material-to-liquid ratio was 1:30). Total phenols were determined as the parameter for assessment, and the levels of individual independent variables for CCD were obtained according to these single factor experiments.

#### 3.2.2. RSM Experiment

After the single factor tests, RSM with CCD was applied to estimate the effect of independent variables (i.e., *X*_1_, extraction temperature; *X*_2_, ultrasonic power; *X*_3_, solvent concentration; *X*_4_, liquid-to-material ratio) and their interactions on UAE of phenolic yield (*Y*_1_) and antioxidant activity (*Y*_2_). Based on preliminary single factor analysis and literature data, levels of independent parameters were selected and coded at five levels according to Equation (1):

(1) $$x_{i}=\frac{X_{i}-X_{0}}{\Delta X_{i}}\;\;\;i=1,2,3,4,$$

where *x_i_* and *X_i_* are the coded and actual values of an independent variable, respectively. *X*_0_ is the actual value on the center point of *X_i_*, and *ΔX_i_* is the value of the step change. The design values of independent variables and their coded values are represented in Table 4. In the present study, CDD conducted 30 experimental points including six replicates at the central point, sixteen factorial points and eight axial points for a full factorial design to study the effect of independent variables on the response. The experiments were randomized and the response values in each trial were analyzed using Design-Expert (Version 8.0.6, Stat-Ease Inc., Minneapolis, MN, USA) and fitted to a second-order polynomial regression model expressing mathematical relationship between independent variables (*X*_1_, *X*_2_, *X*_3_ and *X*_4_) and responses (*Y*_1_ and *Y*_2_):

(2) $$Y=\beta_{0}+\;\sum_{i=1}^{4}\beta_{i}X_{i}+\sum_{i=1}^{3}\sum_{j=i+1}^{4}\beta_{ij}X_{i}X_{j}+\;\sum_{i=1}^{4}\beta_{ii}X_{i}^{2},$$

where, *Y* (*Y*_1_ or *Y*_2_) is the predicted response, *X_i_* and *X*_j_ are diverse input variables that influence the response variable *Y*, *β*_0_ is the constant coefficient, *β_i_* is the linear coefficient, *β_ij_* is the interaction coefficient of two factors (*X*_i_ and *X*_j_), and *β_ii_* is the quadratic coefficient of one factor (*X_i_*^2^).

The obtained model was verified by comparing the phenolic yield and antioxidant ability of the ATL extract obtained under the optimal UAE conditions to those predicted by the models. Furthermore, the resulting solution was analysed by UPLC-QTOF-MS/MS to determine its phenolic composition.

### 3.3. Measurement of Total Phenols

Total phenols were determined according to the Folin–Ciocalteu method [68] with slight modifications. In brief, 40 μL of 25% Folin–Ciocalteu solution was added to designated wells of a 96-well microplate, followed by addition of 20 μL standard (10–400 mg/L gallic acid dissolved and diluted with 50% ethanol, *R*^2^ = 0.999), ATL extract solution or blank (MilliQ water). After blending, 140 μL of 700 mM Na_2_CO_3_ solution was added to each well, and the plate was shaken for 5 min at 250 rpm and incubated in dark at 40 °C for 30 min, followed by absorbance measurement at 765 nm with a microplate reader (xMark™ Microplate Absorbance Spectrophotometer, Bio-Rad, Hercules, CA, USA). Results were expressed as mg gallic acid equivalent (GAE)/100 g d.w. of ATL powder.

### 3.4. Determination of DPPH• Scavenging Activity

Assays of DPPH• scavenging activity were performed using the method described by Alañón et al. [69] with slight modifications. In brief, 10 μL standard (10–400 mg/L Trolox dissolved and diluted with 50% ethanol, *R*^2^ = 0.998), ATL extract solution or blank was added to a designated well of a 96-well microplate. Subsequently, 40 μL of 1 mM freshly prepared DPPH solution was added to each well followed by addition of 190 μL methanol, and the plate was then stood in an orbital shaker setting at 200 rpm for 1 min. After 30 min incubation at room temperature in dark, absorbance was recorded at 517 nm using the microplate reader. Results were expressed as μmol Trolox equivalent (TE)/100 g d.w. of ATL powder.

### 3.5. UPLC-QTOF-MS/MS Analyses of Phenolic Compositions

The UPLC-QTOF-MS/MS system was comprised of an Acquity Ultra-Performance Liquid Chromatography (UPLC) system (Waters, Milford, MA, USA) and a QTOF-MS mass spectrometer (Xevo G2-XS, Waters). A C_18_ column (Diamonsil C_18_ 5 μm 250 × 4.6 mm i.d., Dikma, Beijing, China) was used for separation, and the column temperature was set at 30 °C. The mobile phase was consisted of water with 0.4% formic acid (*v*:*v*) (A) and acetonitrile (B) under the following gradient program: 0–10 min, 10% B; 10–12 min, 10%–18% B; 12–33 min, 18% B; 33–35 min, 18%–15% B; 35–40 min, 15% B; 40–42 min, 15%–18% B; 42–60 min, 18% B; 60–80 min, 18%–50% B. The flow rate was set at 1 mL/min with an injection volume of 10 μL. Mass spectra were recorded in the range of *m*/*z* 100–1500. MS experiments were performed both in positive and negative ionization mode under the following conditions: nitrogen drying gas flow, 10.0 L/min; nebulizer pressure, 45 psi; gas drying temperature, 370 °C; capillary and fragmentor voltage, 2.500 kV; and with MS/MS collision energies set at 20 V. Peak identification was performed by comparing the mass spectra and fragmentation ions with data from reported literatures.

### 3.6. Statistical Analysis

Experiment design was performed using Design-Expert. All experimental results obtained were expressed as means ± SD, and data were analyzed by analysis of variance (*p* < 0.05) using SPSS software (Version 22.0, SPSS Inc., Chicago, IL, USA). All analyses were performed in triplicate.

## 4. Conclusions

The current study is the first report on the effect of ultrasonic stimulation on the phenolic yield and antioxidant activity of ATL. A quadratic model fitted well both responses, i.e., total phenol yield and antioxidant activity. Operating parameters were optimized using single factor experiments and a response surface design. Optimal conditions were found to be percentage of aqueous ethanol 66.21%, material-to-liquid ratio 1:15.25 g/mL, extraction temperature 60 °C; ultrasonic power 270 W, and ultrasound time 30 min, which gave a maximum total phenol yield of 7593.62 mg GAE/100 g d.w. and antioxidant activity of 74,241.61 μmol TE/100 g d.w. The verified results confirmed the adequate fit of the predicted model. Furthermore, 22 phenolic compounds were discovered in the present study with UPLC-QTOF-MS/MS analysis, which indicated the superiority of the extraction conditions and provided new insights into the phenolic components of ATL. All this information should be useful for both researchers and consumers, and contribute to the better utilization of ATL as a source of phenolic compounds with high antioxidant activity.

## Figures and Tables

**Table 1:** Response surface central composite design and experimental data and predicted values for extraction yield of phenols in ATL.

Run	Factors				Total Phenols (mg/100 g)	DPPH (μmol/100 g)	DPPH Activity/Total Phenols (μmol/mg)
	*X*_1_	*X*_2_	*X*_3_	*X*_4_	Experimental (Predicted) Values		
	C (%)	R (g/L)	P (W)	T (°C)			
1	70(0)	20(0)	240(0)	50(0)	7467.83 (7449.86)	71,695.05 (72,589.38)	9.60
2	60(−1)	15(−1)	270(1)	60(1)	7591.30 (7534.35)	70,380.34 (70,880.44)	9.27
3	70(0)	20(0)	240(0)	50(0)	7417.39 (7449.86)	73,356.78 (72,589.38)	9.89
4	80(1)	25(1)	270(1)	40(−1)	6622.83 (6632.18)	57,161.41 (56,161.13)	8.63
5	80(1)	25(1)	210(−1)	60(1)	6840.22 (6732.65)	51,733.24 (52,828.09)	7.56
6	80(1)	15(−1)	270(1)	60(1)	7005.87 (6927.40)	66,300.47 (65,584.34)	9.46
7	60((−1)	15(−1)	210(−1)	40(−1)	7281.30 (7204.18)	64,060.73 (63,443.39)	8.80
8	60(−1)	25(1)	270(1)	40(−1)	7180.98 (7062.72)	60,133.24 (61,057.29)	8.37
9	70(0)	20(0)	240(0)	70(2)	7384.35 (7425.71)	63,223.73 (62,238.86)	8.56
10	60(−1)	15(−1)	270(1)	40(−1)	7158.91 (7192.11)	60,282.07 (59,038.22)	8.42
11	60(−1)	25(1)	210(−1)	40(−1)	7168.48 (7172.58)	68,041.95 (68,609.08)	9.49
12	70(0)	20(0)	240(0)	30(−2)	7037.83 (7125.72)	56,420.30 (57,524.26)	8.02
13	70(0)	20(0)	240(0)	50(0)	7256.52 (7449.86)	70,844.41 (72,589.38)	9.76
14	50(−2)	20(0)	240(0)	50(0)	6776.52 (6846.72)	57,567.32 (56,833.36)	8.50
15	80(1)	15(−1)	210(−1)	40(−1)	6609.78 (6505.13)	58,025.57 (56,190.23)	8.78
16	80(1)	25(1)	210(−1)	40(−1)	6772.83 (6774.90)	60,425.89 (59,955.71)	8.92
17	90(2)	20(0)	240(0)	50(0)	5783.04 (5842.09)	42,030.84 (42,883.89)	7.27
18	70(0)	10(−2)	240(0)	50(0)	7026.96 (7141.37)	66,619.14 (67,862.93)	9.48
19	70(0)	20(0)	300(2)	50(0)	7106.09 (7147.63)	69,835.50 (68,702.84)	9.83
20	60(−1)	25(1)	270(1)	60(1)	7148.64 (7178.92)	62,292.05 (63,978.39)	8.71
21	80(1)	15(−1)	210(−1)	60(1)	6625.54 (6688.92)	58,877.87 (57,983.73)	8.89
22	70(0)	20(0)	180(−2)	50(0)	6931.30 (7019.01)	67,402.26 (68,654.02)	9.72
23	70(0)	20(0)	240(0)	50(0)	7519.13 (7449.86)	72,961.25 (72,589.38)	9.70
24	70(0)	20(0)	240(0)	50(0)	7587.83 (7449.86)	72,829.01 (72,589.38)	9.60
25	70(0)	20(0)	240(0)	50(0)	7450.43 (7449.86)	73,849.78 (72,589.38)	9.91
26	80(1)	25(1)	270(1)	60(1)	6851.09 (6873.33)	56,634.84 (57,282.08)	8.27
27	70(0)	30(2)	240(0)	50(0)	7040.87 (7055.71)	65,851.05 (64,726.36)	9.35
28	80(1)	15(−1)	270(1)	40(−1)	6513.91 (6460.19)	54,193.49 (55,542.27)	8.32
29	60(−1)	25(1)	210(−1)	60(1)	7006.52 (7005.36)	64,600.48 (63,281.61)	9.22
30	60(−1)	15(−1)	210(−1)	60(1)	7346.74 (7263.01)	66,185.78 (67,037.04)	9.01

**Table 2:** Analysis of variance for the extraction of phenols yield (*Y*_1_) and antioxidant capacity (*Y*_2_).

	Source	Coefficient Estimate	Sum of Squares	Degree of Freedom	Standard Error	Mean Square	*F*-Value	*p*-Value
*Y*_1_	Model	7449.86	4.16 × 10^6^	14	43.66	2.97 × 10^5^	25.95	<0.0001 **
	*X*_1_	−251.16	1.51 × 10^6^	1	21.83	1.51 × 10^6^	132.36	<0.0001 **
	*X*_2_	−21.42	1.10 × 10^4^	1	21.83	1.10 × 10^4^	0.96	0.3422
	*X*_3_	32.15	2.48 × 10^4^	1	21.83	2.48 × 10^4^	2.17	0.1614
	*X*_4_	75	1.35 × 10^5^	1	21.83	1.35 × 10^5^	11.8	0.0037 **
	*X*_1_*X*_2_	75.34	9.08 × 10^4^	1	26.74	9.08 × 10^4^	7.94	0.0130 *
	*X*_1_*X*_3_	−8.22	1.08 × 10^3^	1	26.74	1.08 × 10^3^	0.09	0.7628
	*X*_1_*X*_4_	31.24	1.56 × 10^4^	1	26.74	1.56 × 10^4^	1.37	0.2609
	*X*_2_*X*_3_	−24.45	9.56 × 10^3^	1	26.74	9.56 × 10^3^	0.84	0.375
	*X*_2_*X*_4_	−56.51	5.11 × 10^4^	1	26.74	5.11 × 10^4^	4.47	0.0517
	*X*_3_*X*_4_	70.85	8.03 × 10^4^	1	26.74	8.03 × 10^4^	7.02	0.0182 *
	*X*_1_^2^	−276.36	2.09 × 10^6^	1	20.42	2.09 × 10^6^	183.16	<0.0001 **
	*X*_2_^2^	−87.83	2.12 × 10^5^	1	20.42	2.12 × 10^5^	18.5	0.0006 **
	*X*_3_^2^	−91.63	2.30 × 10^5^	1	20.42	2.30 × 10^5^	20.14	0.0004 **
	*X*_4_^2^	−43.54	5.20 × 10^4^	1	20.42	5.20 × 10^4^	4.55	0.05
	Residual		1.72 × 10^5^	15		1.14 × 10^4^		
	Lack of Fit		1.09 × 10^5^	10		1.09 × 10^4^	0.87	0.6032
	Pure Error		6.26 × 10^4^	5		1.25 × 10^4^		
	Cor Total		4.33 × 10^6^	29				
	*R*^2^	0.9604						
	Adj *R*^2^	0.9233						
	Pred *R*^2^	0.8341						
	Adeq Precision	22.3775						
	C.V. %	1.5169						
	*r*	0.98						
*Y*_2_	Model	72,589.38	1.57 × 10^9^	14	608.6	1.12 × 10^8^	50.34	<0.0001 **
	*X*_1_	−3487.37	2.92 × 10^8^	1	304.3	2.92 × 10^8^	131.34	<0.0001 **
	*X*_2_	−784.14	1.48 × 10^7^	1	304.3	1.48 × 10^7^	6.64	<0.0001 **
	*X*_3_	12.2	3.57 × 10^3^	1	304.3	3.57 × 10^3^	0	0.0210 *
	*X*_4_	1178.65	3.33 × 10^7^	1	304.3	3.33 × 10^7^	15	0.9685
	*X*_1_*X*_2_	−350.05	1.96 × 10^6^	1	372.69	1.96 × 10^6^	0.88	0.0015 **
	*X*_1_*X*_3_	939.3	1.41 × 10^7^	1	372.69	1.41 × 10^7^	6.35	0.3625
	*X*_1_*X*_4_	−450.04	3.24 × 10^6^	1	372.69	3.24 × 10^6^	1.46	0.0235 *
	*X*_2_*X*_3_	−786.65	9.90 × 10^6^	1	372.69	9.90 × 10^6^	4.46	0.2459
	*X*_2_*X*_4_	−2230.28	7.96 × 10^7^	1	372.69	7.96 × 10^7^	35.81	0.052
	*X*_3_*X*_4_	2062.14	6.80 × 10^7^	1	372.69	6.80 × 10^7^	30.62	<0.0001 **
	*X*_1_^2^	−5682.69	8.86 × 10^8^	1	284.65	8.86 × 10^8^	398.56	<0.0001 **
	*X*_2_^2^	−1573.68	6.79 × 10^7^	1	284.65	6.79 × 10^7^	30.56	<0.0001 **
	*X*_3_^2^	−977.74	2.62 × 10^7^	1	284.65	2.62 × 10^7^	11.8	<0.0001 **
	*X*_4_^2^	−3176.95	2.77 × 10^8^	1	284.65	2.77 × 10^8^	124.57	0.0037 **
	Residual		3.33 × 10^7^	15		2.22 × 10^6^		<0.0001 **
	Lack of Fit		2.71 × 10^7^	10		2.71 × 10^6^	2.18	0.2015
	Pure Error		6.22 × 10^6^	5		1.24 × 10^6^		0.2015
	Cor Total		1.60 × 10^9^	29				
	*R*^2^	0.9792						
	Adj *R*^2^	0.9597						
	Pred *R*^2^	0.8968						
	Adeq Precision	28.1801						
	C.V. %	2.3491						
	*r*	0.9898						

* 0.01 ≤ *p* < 0.05; ** *p* < 0.01.

**Table 3:** Phenolic compounds tentatively identified in ATL by UPLC-QTOF-MS/MS analyses.

Peak	*R*_t_ (min)	[M − H]^−^ (*m*/*z*)	Error (ppm)	Formula	MS/MS Fragments *m*/*z* (% Base Peak)	Proposed Compound
1	2.35	191.0564	4.2	C_7_H_11_O_6_	127.0405 (3.7), 111.0453 (1.2)	Quinic acid
2	4.03	343.0654	−3.2	C_14_H_15_O_10_	191.0559 (28.0), 169.0139 (100.0), 125.0243 (73.3)	Theogallin
3	4.29	169.0143	3.5	C_7_H_5_O_5_	125.0244 (100), 169.0139 (74.1)	Gallic acid
4	6.91	353.0864	−2.5	C_16_H_17_O_9_	191.0552 (100.0), 179.0348 (53.0), 135.0449 (69.3)	3-*O*-Caffeoylquinic acid
5	7.85	353.0862	−3.1	C_16_H_17_O_9_	191.0550 (100), 179.0339 (54.7), 135.0444 (68.0)	5-*O*-Caffeoylquinic acid
6	11.05	285.0612	0.7	C_12_H_13_O_8_	153.0177 (25.6), 109.0272 (17.5)	Uralenneoside
7	12.64	337.0918	−1.5	C_16_H_17_O_8_	191.0550 (47.6), 163.0393 (100.0), 119.0494 (65.8)	*cis*-4-*p*-Coumaroylquinic acid
8	12.93	337.0923	0	C_16_H_17_O_8_	191.0558 (19.4), 163.0392 (100.0), 119.0498 (54.1)	*cis*-5-*p*-Coumaroylquinic acid
9	14.4	183.0323	16.4	C_8_H_7_O_5_	183.0300 (31.4), 124.0194 (100.0)	4-*O*-Methyl-gallate
10	16.08	755.2031	−0.5	C_33_H_39_O_20_	609.1443 (54.7), 463.2144 (11.2), 301.0345 (21.1)	Quercetin-3-*O*-rhamninoside
11	16.39	289.0717	1.7	C_15_H_13_O_6_	245.0822 (32.9), 211.0291 (7.7)	(+)-Catechin
12	16.91	863.1819	−0.5	C_45_H_35_O_18_	289.0710 (78.7)	cinnamtannin B1
13	18.65	479.0822	−0.8	C_21_H_19_O_13_	316.0219 (100.0), 287.0192 (16.2), 271.0237 (30.5)	Myricetin-*O*-hexoside I
14	19.01	479.0828	0.4	C_21_H_19_O_13_	316.0221 (100.0), 271.0249 (28.5)	Myricetin-*O*-hexoside II
15	19.38	593.1513	1.2	C_27_H_29_O_15_	593.1505 (100.0), 447.0915 (53.1), 301.0343 (61.8)	Quercetin-3,7-*O*-α-l-dirhamnopyranoside
16	21.47	787.1001	0.9	C_34_H_27_O_22_	615.0979 (18.8), 465.0670 (15.3), 169.0137 (22.7)	1,2,3,6-Tetrakis-*O*-galloyl-β-D-glucose
17	21.95	449.0727	1.6	C_20_H_17_O_12_	316.0228 (100.0), 271.0247 (37.8)	Myricetin-arabinoside/xylopyranoside Isomer
18	23.1	463.0878	0.2	C_21_H_19_O_12_	316.0218 (100.0), 287.0194 (18.9)	Myricitrin
19	24.15	463.0877	0	C_21_H_19_O_12_	300.0267 (100.0), 255.0293 (20.9)	Quercetin-3-*O*-galactoside
20	25.09	463.0884	1.5	C_21_H_19_O_12_	300.0273 (100.0), 255.0297 (22.6)	Quercetin-3-*O*-glucoside (isoquercetin)
21	26.51	939.1124	2.1	C_41_H_31_O_26_	769.0892 (57.3), 617.0776 (20.1), 447.0559 (9.6), 169.0140 (94.8)	Pentagalloyl glucose isomer
22	28.23	939.1109	0.5	C_41_H_31_O_26_	769.0883 (51.5), 617.0775 (19.2), 447.0550 (9.1), 169.0140 (51.2)	Pentagalloyl glucose isomer
23	30.12	433.0768	−0.7	C_20_H_17_O_11_	300.0271 (100.0), 255.0286 (26.8), 243.0291 (10.4)	Quercetin-3-*O*-arabinopyranoside
24	33.32	433.0767	−0.9	C_20_H_17_O_11_	300.0266 (100.0), 271.0236 (59.7), 255.0297 (35.1)	Quercetin 3-*O*-arabinofuranoside
25	34.94	447.0927	0	C_21_H_19_O_11_	300.0270 (100.0), 271.0249 (51.2), 255.0295 (27.3)	Quercetin 3-*O*-rhamnoside
26	56.11	1091.12	−1	C_48_H_35_O_30_	939.1072 (60.0), 769.0875 (22.8), 169.0133 (100.0)	Hexagalloyl glucose
27	60.3	431.0974	−0.9	C_21_H_19_O_10_	285.0387 (100.0), 255.0288 (59.4), 227.0336 (34.9)	Kaempferol-3-*O*-α-l-rhamnoside
28	69.26	609.1234	−1.6	C_30_H_25_O_14_	463.0871 (68.8), 300.0274 (95.3)	Quercetin-3-*O*-rutinoside
29	71.72	301.0354	2	C_15_H_9_O_7_	301.0344 (45.7), 243.0662 (100.0)	Quercetin

**Table 4:** Independent variables and their levels and corresponding coded values used in CCD.

Independent Variables	Independent Levels				
	−2	−1	0	1	2
Solvent concentration, *X*_1_ (%)	50	60	70	80	90
Material-to-liquid ratio, *X*_2_ (g/mL)	1:5	1:15	1:20	1:25	1:30
Extraction temperature, *X*_3_ (°C)	30	40	50	60	70
Sonication power, *X*_4_ (W)	180	210	240	270	300
